# Sensitive and Selective Theophylline Electrochemical Detection on ZnO Nanoflowers/Graphene Oxide/Boron-Doped Diamond Nanoparticles

**DOI:** 10.1021/acsomega.5c03180

**Published:** 2025-06-20

**Authors:** Syarifa Sabilla, Prastika Krisma Jiwanti, Takeshi Kondo, Rena Akiyama, Taisuke Kusunoki, Afif Akmal Afkauni, Anisa Sufia Latifah, Arramel, Tahta Amrillah, Yeni Wahyuni Hartati, Qonita Kurnia Anjani, Yulia Mariana Tesa Ayudia Putri, Jarnuzi Gunlazuardi

**Affiliations:** † Nanotechnology Engineering, Faculty of Advanced Technology and Multidiscipline, Universitas Airlangga, Surabaya 60115, Indonesia; ‡ Airlangga Functional Nanomaterials Research Group, Faculty of Advanced Technology and Multidiscipline, Universitas Airlangga, Surabaya 60115, Indonesia; § Department of Pure and Applied Chemistry, Tokyo University of Science, 2641 Yamazaki, Noda, Chiba 278-8510, Japan; ∥ Center of Excellence Applied Physics and Chemistry, Nano Center Indonesia, South Tangerang, Banten 15314, Indonesia; ⊥ Department of Chemistry, Faculty of Mathematics and Natural Sciences, Universitas Padjadjaran, Jatinangor 45363, Indonesia; # School of Pharmacy, Medical Biology Centre, Queen’s University Belfast, 97 Lisburn Road, Belfast BT9 7BL, U.K.; ∇ Department of Chemistry, Faculty of Mathematics and Natural Sciences, Universitas Indonesia, Kampus UI, Depok, Jakarta 16424, Indonesia

## Abstract

Theophylline is among bronchodilator drugs consumed to overcome breathing difficulties, but accurate monitoring is required to avoid side effects. Therefore, this study aimed to develop a screen-printed electrode (SPE). Theophylline is among bronchodilator drugs consumed to overcome breathing difficulties, but accurate monitoring is required to avoid side effects. Therefore, this study aims to develop a screen-printed electrode (SPE) modified with zinc oxide nanoflowers (ZnONF) and graphene oxide (GO) nanocomposites along with boron-doped diamond nanoparticles (ZnONF/GO/BDDNPs/SPE) to detect theophylline. ZnONF is synthesized using the hydrothermal method, and the nanoflower morphology is confirmed through field emission-scanning electron microscopy with an average petal size of 62.33 nm. In electrochemical detection of theophylline, the differential pulse voltammetry (DPV) technique is used to determine the signal to background (S/B), linearity and detection limits, selectivity, reproducibility, and recovery. The results show that the S/B value produced by the ZnONF/GO/BDDNPs/SPE electrode is 4.91. The ZnONF/GO/BDDNPs/SPE electrode also exhibits the highest active surface area (0.045 cm^2^), facilitating enhanced electron transfer. This value is higher compared to that of the ZnONF unmodified electrode. The limits of detection and quantification values are 0.17 and 0.58 μM, respectively. Theophylline measurements using the electrodes prove to be selective against similar compounds and determination in the real sample, namely, theobromine, caffeine, ascorbic acid, and uric acid. Good precision values are obtained with a %RSD value of 0.73%. The accuracy is tested in actual samples of urine, medicine, and coffee, indicating good recovery values of 103.69, 96.2, and 94.19%, respectively. In conclusion, ZnONF/GO/BDDNPs electrode is suitable for theophylline determination and health monitoring.

## Graphical abstract

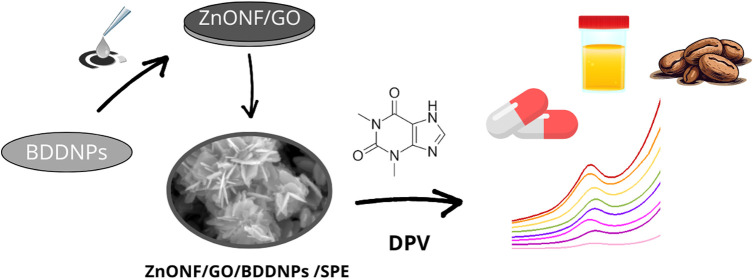

## Introduction

Theophylline (1,3-dimethylxanthine) is a natural alkaloid often used as a bronchodilator and respiratory stimulant medication for chronic breathing difficulties, asthma, bronchiectasis, chronic obstructive pulmonary disorder, neonatal apnea, and emphysema.^1^ The performance of this drug starts on entering the body, causing the relaxation of muscles which makes breathing easier.^2^ For effective treatment, the normally accepted blood concentration of theophylline is 10–20 μg/mL for asthma and 6–11 μg/mL for neonatal apnea. Concentrations below 10 μg/mL will cause mild effects such as nausea, headache, and anxiety, while those above 20 μg/mL lead to more serious side effects including tremors, agitation, insomnia, diarrhea, palpitations, cardiac arrhythmias, and seizures.^3^ To avoid these side effects, it is necessary to accurately detect the intake levels of theophylline in the body.

Several analytical methods are used, such as high-performance liquid chromatography (HPLC), spectrometry, and radioimmunoassay, to detect theophylline in samples. Although chromatography and spectrometry methods are widely used for quantitative analysis, there are several limitations, such as expensive and complicated instrumentation tools, difficult sample preparation, expensive solvents, and high levels of toxicity. In some cases, low selectivity and sensitivity were also reported.^4–6^ In addition, radioimmunoassay methods also have limitations such as radioactive compounds, expensive laboratory, and technician settings.^7^ Therefore, relatively inexpensive, sensitive, selective, and environmentally friendly analytical methods are needed. Electrochemical methods offer many advantages over other analytical methods including high sensitivity and selectivity, easy to use, simple and compact, portability, relatively inexpensive, simple operating methods, and applicable for small sample requirements.^8,9^ In this study, the screen-printed electrode (SPE) was used because it has a wide potential window and low background current depending on the material of the working electrode used, and the surface can also be easily modified.^10^ In the electrochemical method, the use of bare electrodes causes problems, such as slow electron transfer and easy electrode fouling. These weaknesses can be overcome by performing modifications such as nanomaterials required in electron transmission, improved sensitivity, and selectivity that make good contributors to electrochemical reactions. Currently, carbon materials and semiconductor materials make a major contribution as suitable electrode modifications in the manufacture of efficient electrochemical sensors, due to their ability to enhance electrical conductivity and provide a large surface area.^11,12^

Boron-doped diamond nanoparticles (BDDNPs) are potential materials in electrochemical sensors due to the low background currents, wide potential window of 3.5 V, as well as good chemical and physical stability.^12–16^ Several significant advantages of metal-modified BDDNPs electrodes were introduced in the earlier investigation including high sensitivity, low background current, low limit of detection (LOD) values, reversible electron transfer kinetics, as well as long-term stability.^17^ On the other hand, graphene oxide (GO) is an oxidized derivative of graphene with several functional groups such as hydroxyl, epoxy, carbonyl, and carboxyl. GO has a high surface area, is cheap, and easy to produce, making it a good candidate for sensor modification.^17–19^ It also has other advantages including being easy to obtain, high thermal and chemical stability, as well as dissolve in water and other polar solvents.^20^ Aside from BDDNPs and GO, zinc oxide (ZnO) is a semiconductor nanomaterial with a large excitation binding energy of 60 meV and a wide band gap of 3.37 eV. It also has good electrical conductivity, low toxicity, widely available, high chemical and thermal stability, easy to synthesize at low cost, and good biocompatibility.^21^ Furthermore, ZnO has been used in electrode fabrication for sensors due to its superior electrocatalytic properties. The nanomaterial can be synthesized through various bottom-up methods. In particular, hydrothermal is a versatile method that inserts precursor solution into special vessels.^22,23^ The advantages include being simple, short preparation time, and small size distribution.^24^ A peculiar outcome of this method is to facilitate the nanoflowers formation, which offers many active sites on the surface for analyte adsorption.^25^ ZnO nanoflowers (ZnONF) composite to the GO layer may provide several advantages including increased surface area due to the synergistic effect between ZnONF and GO, better electrical conductivity, and increased electron transfer rate 10. ZnONF/GO has shown interesting catalytic activity in electrochemical applications.^26^ The nanocomposites also demonstrated good sensitivity, selectivity, and response time.^27^

The SPE used is a commercial product with carbon as the working electrode and auxiliary electrode, while Ag/AgCl was the reference. This study used SPE modified with ZnONF/GO/BDDNPs as a working electrode to detect theophylline. The novelty of this study lies in the synthesis of ZnONF/GO/BDDNPs nanocomposite-modified electrodes that are easy to synthesize, cost-effective, have a high surface area, and exhibit catalytic activity in the detection of theophylline. The use as a modifier results in an interesting electrochemical response and has the potential for use beyond theophylline detection.

## Experimental Section

### Chemicals and Reagents

Theophylline (≥99%), graphene oxide (≥98%), theobromine (≥98%), sodium dodecyl sulfate (≥99%), ascorbic acid (≥99%), and uric acid (≥99%) were procured from Sigma-Aldrich. ZnSO4 (≥99%) was purchased from Merck. Ethanol, potassium hexacyanoferrat (≥99%), and potassium chloride (≥99.5%) were purchased from Supelco. BDDNPs powder size 0–250 nm was acquired from Hunan Boromond EPT Co. Ltd. with boron doping levels of 300–1000 ppm. Caffeine (≥99%) was obtained from Nitra Kimia, Surabaya, Indonesia. Theophylline capsules branded Theobron, coffee, and healthy human urine were used for the selectivity measurement. The phosphate buffer solution (PBS) was prepared by the combination of two chemical solutions, namely, Na_2_HPO_4_·H_2_O and NaH_2_PO_4_·H_2_O mixed in deionized water (DI). PBS solution was used as an electrolyte for the sensing experiments. Furthermore, pH was measured with a pH meter (pH 7), and DI water was used for all of the electrochemical experiments. All chemicals were used without further purification.

### Synthesis of ZnONF/GO

ZnONFs were synthesized using hydrothermal methods. About 0.2 M zinc sulfate powder was added to a 0.8 M NaOH solution. After constant stirring, the solution turned white and was transferred to a Teflon beaker covered by a stainless-steel autoclave kept at 160 °C for 12 h. The sample was centrifuged and washed several times using DI water and ethanol followed by drying at 60 °C to obtain a white powder of ZnONF.^21^ GO was dissolved in 50 mL of sodium dodecyl sulfate (5 mg/mL), and then ZnONF was added in a ratio of 1:1 to GO. The sample was stirred and sonicated to produce a diuniform dispersion followed by centrifugation and washing several times using DI water and ethanol. The sample was dried at 60 °C to obtain a white powder of ZnONF/GO.^28^ Morphology was characterized using FESEM, and then ZnONF, GO, and ZnONF/GO samples were characterized using ultraviolet–visible spectroscopy (UV–vis) (Thermo Scientific Orion Aquamate 8100), and transmission electron microscopy (TEM) (JEM-2100F, Jeol). In addition, X-ray diffraction (XRD) (Rigaku MiniFlex 600-C), attenuated total reflectance-Fourier transform infrared (ATR-FTIR) (Thermo Scientific Nicolet iS10), and DXR3i Raman imaging spectroscopy (Thermo Fisher Scientific Raman) equipped with a 532 nm laser excitation source at room temperature were used. The parameters used include laser power of 5.5 mW, exposure time of 0.02000 s (50 Hz), and 50 scans with the Raman shift range of 50–3400 cm^–1^.

### Preparation of the Working Electrode and Measurement Procedures

The screen-printed electrode (SPE) used is a commercial product of TailKuKe with carbon as the working electrode and auxiliary electrode, while Ag/AgCl was the reference. The freshly synthesized sample was used for electrochemical measurements by modifying the working electrode. Modification of BDDNPs on SPE surfaces (BDDNPs/SPE) started with the preparation of ink BDDNPs. About 10 mg BDDNPs were added to 0.5 mL of 30 wt % ethanol and dispersed by ultrasonication, then 6 μL were drop-cast on the SPE surface and dried.

Modification of GO/BDDNPs/SPE started with 10 mg of GO added to 0.5 mL of ethanol 30 wt % and dispersed by ultrasonication. Subsequently, 6 μL of GO was drop-cast on the surface of SPE coated with BDDNPs and dried. Modification of ZnONF/GO/BDDNPs/SPE started with 10 mg of ZnONF/GO added to 0.5 mL of ethanol 30 wt % and dispersed by ultrasonication. About 6 μL of ZnONF/GO was drop-cast on the surface of SPE coated with BDDNPs and dried. The modified electrode was used for the electrochemical investigation as a working electrode. Furthermore, the three electrodes prepared were characterized using scanning electron microscopy-energy-dispersive X-ray spectroscopy (SEM-EDX) (JSM-7600F, JEOL).

Cyclic voltammetry (*C–V*) was used in measurements with a potential range of −0.4 to +0.7 V. Electrochemical impedance spectroscopy (EIS) was used in measurements with a frequency range from 0.1 Hz to 100 kHz. In the *C–V* and EIS analysis, a 5 mM K_3_[Fe­(CN)_6_] solution combined with 0.1 M KCl was used. Differential pulse voltammetry (DPV) was used in measurements with a potential range of 0 to +1.5 V, step potential of 0.12 V, pulse potential of 0,1 V, scan rate of 0.12 V/s, and pulse duration of 0.05 V. About 120 μM theophylline from stock solution (1 mM) was added to 0.1 M PBS at pH 7 as a supporting electrolyte. Solutions having a similar chemical structure were prepared to analyze the effect of interference on the electrochemical performance of theophylline and transferred to electrochemical cells in a ratio of 1:1 v/v with each compound at a concentration of 120 μM. All experimental measurements were made at room temperature using Emstat^3+^ Blue Palmsens Potentiostat.

### Preparation of Real Samples

Urine samples were obtained from healthy volunteers; then, 120 μM theophylline was added to prepare a spiked preparation. Other real samples used were theophylline capsules obtained from pharmacies and commercially available coffee powder obtained from convenience stores. A small amount of powder in theophylline capsules that was dissolved and diluted to a concentration of 1 mM was used as a stock solution in DI water. Subsequently, 120 μM theophylline was added to 0.1 M PBS pH 7. A stock solution of coffee powder dissolved in DI water with a concentration of 0.1 g/mL was prepared, and then 120 μM theophylline was added. The exact concentration of the real samples was checked by using the DPV technique. The amount of theophylline in the real sample was determined by using a calibration plot. Based on DPV technique measurements, theophylline recovery in real samples was estimated.

## Results and Discussion

### Material Characterization

The absorption spectra of GO, ZnONF, and ZnONF/GO synthesis materials were collected between absorption and wavelength and examined through a UV–vis spectrophotometer (Figure 1). The GO absorption peak near 288 nm was reflected in the n-π* transitions of aromatic C=O bonds (Figure S1).^29^ ZnONF showed a sharp band at 374 nm, confirming the absorption peak characteristic of the hexagonal phase of wurtzite.^30^ The UV–vis absorbance of ZnONF/GO was found at the same wavelength of 374 nm, which can be attributed to the intrinsic band gap absorption of ZnONF.^29^

**Figure 1:**
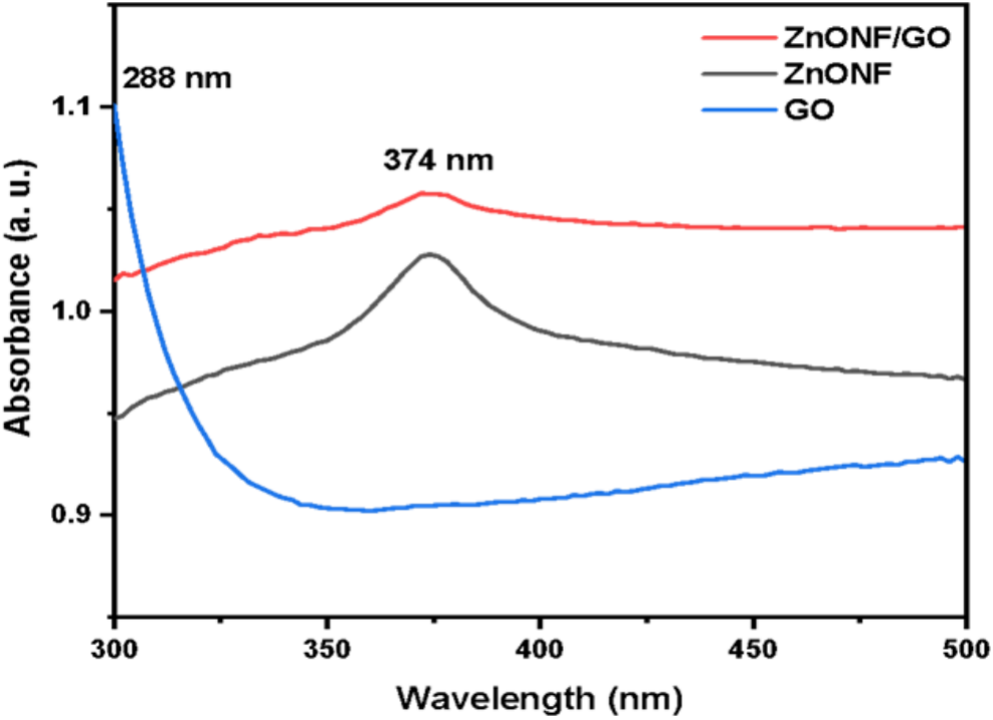
UV–vis spectra of ZnONF, GO, and ZnONF/GO.

Raman spectroscopy is one of the most powerful methods to determine the allotrope of carbon-based materials due to the ability to probe the microstructure of carbon-based materials such as orientation, number of layers, defects, disorder, state of dispersion, and even doping.^30,31^ In this study, Raman spectroscopy along with the FTIR method was used to compare the vibrational properties between GO and ZnONF/GO.

Both samples were observed to display the signature of carbon-based materials, which possess distinct vibrational peaks around 1300 and 1500 cm^–1^ (Figure 2). These peaks are commonly attributed to the D and G bands of carbon allotropes, respectively. D band is usually indicative of defect sites such as vacancies and grain boundaries, while G band is associated with the first-order scattering of E_2g_ phonon found in the sp^2^ bonds.^32–35^ The peak located at around 2600 cm^–1^ is commonly attributed to 2D band of graphene-based materials for both GO and composite.^33,34^

**Figure 2:**
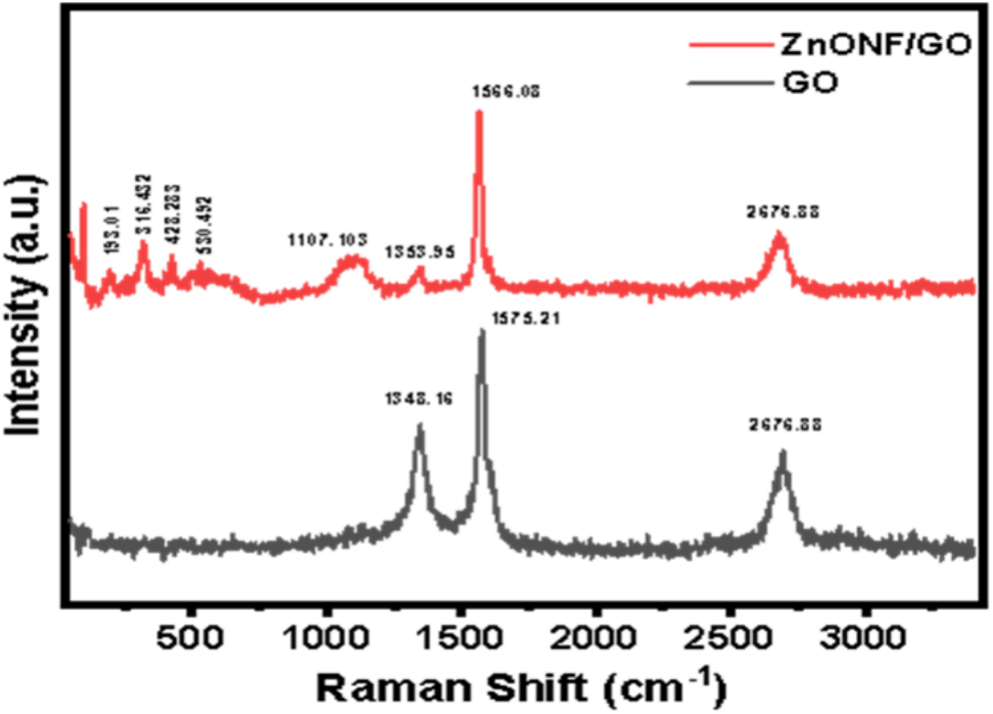
Raman spectra of GO (black) and ZnONF/GO (red) collected at room temperature.

The results showed new peaks upon the incorporation of ZnO into the GO structure. The major peaks around 96, 316, 428, and 530 cm^–1^ can be attributed to the basic phonon modes of hexagonal ZnO, corresponding to E_2L_, A_1_(TO), E_2H_, and A_1_(LO)/E_1_(LO).^36,37^ In addition, the emergent peak at 193 cm^–1^ corresponded to the second-order phonon mode of 2E_2l_, and the peak located at around 1107 cm^–1^ was attributed to the acoustic combination of A_1_ and E_2_.^38^

From the Raman spectra, the graphitization degree of the carbon-based materials can be determined by comparing the intensity between D and G bands, namely, the (ID/IG) ratio. A smaller factor (R) value is attributed to the higher degree of graphitization that correlates to the magnitude of defects and disorder.^33,35^

R=ID/IG

Pristine GO sample has an *R*-value of 0.57, while the ZnONF/GO composite experienced a substantial decrease to 0.13, indicating an ordered structure. The increase in graphitization degree has been proven to show a better electrochemical capacity and charge–discharge performance.^39^

FTIR measurement was performed to determine the functional groups of the synthesized carbon-based materials, as shown in Figure 3. In the case of GO materials, the stretching vibration modes of C–O–C epoxy and C–OH functional groups located were determined at the wavenumber ranges of 600–900 cm^–1^ and 1020–1250 cm^–1^, respectively.^40^ The presence of the aromatic framework of GO materials was found at ∼1427 and 1645 cm^–1^. The carboxyl group (C=O) was detected at 1733 cm^–1^, while narrow peaks at 2909 and 2971 cm^–1^ corresponded to the presence of CH group.^40,41^ The small peak at 3647 cm^–1^ could be attributed to the −OH group originating from the intercalating water within the GO structure. After ZnO was incorporated, the stretching band was observed at the wavenumber of 452 and 667 cm^–1^, indicating successful incorporation into the GO framework.^40,42^

**Figure 3:**
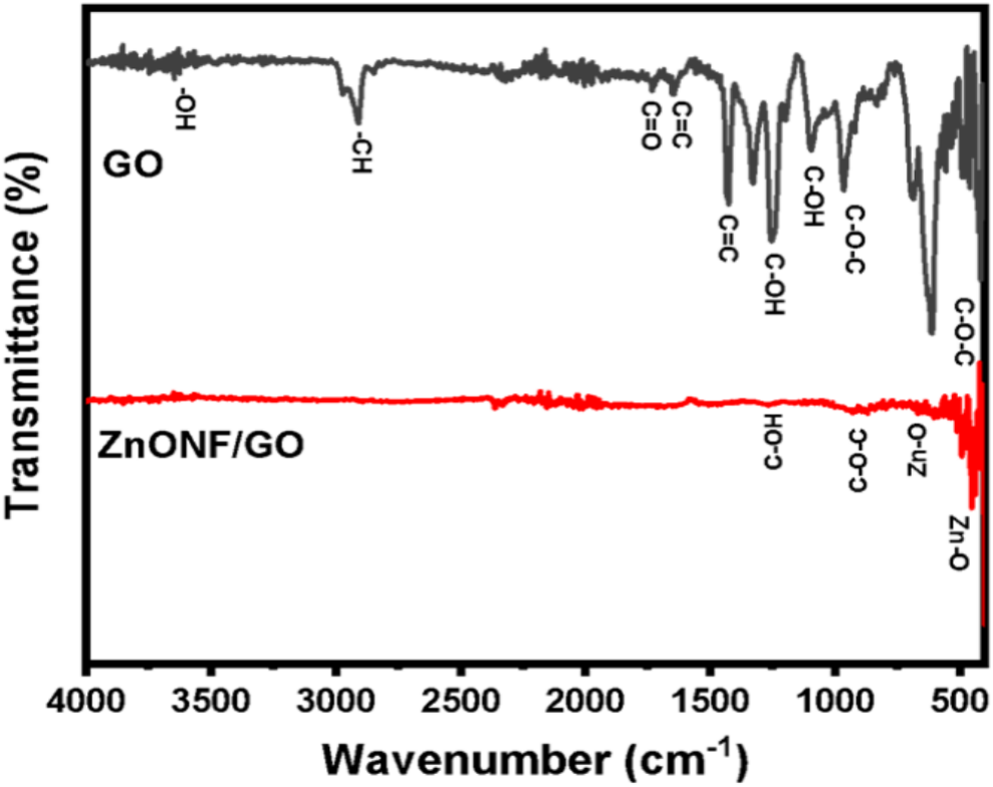
FTIR spectra of GO (black) and ZnONF/GO (red) collected at room temperature.

The XRD spectra of the ZnONF, GO, and ZnONF/GO samples are shown in Figure 4. The XRD GO pattern (black) shows a peak at a 2θ value of 26.32° associated with the reflection of (002). The corresponding peaks obtained indicate that GO has a hexagonal structure.^43^ The ZnONF diffraction patterns (red) were obtained at 31.72, 34.39, 36.21, 47.50, 56.53, 62.75, 66.31, 67.87, 68.97, 72.47, and 76.88° corresponding to the crystal planes (100), (002), (101), (102), (110), (103), (200), (112), (201), (004), and (202) respectively. This result is in agreement with the JCPDS-Card No. 36-1451.^44^ ZnO peaks confirm the properties of crystals and the hexagonal phase structure of wurtzite. The most prominent and sharp peak was found at the (101) and (100) planes, indicating that all the synthesized samples have a good crystalline structure. Moreover, no additional peaks were detected that could be attributed to impurities or transition materials, confirming the purity of the synthesized ZnO nanomaterial. The peaks produced from ZnONF/GO nanocomposites consisted of 25.48° (002) from GO samples as well as 31.74, 34.42, 36.23, 47.52, 56.55, 62.78, 66.30, 67.88, 68.98, 72.51, and 76.89°, indicating peaks of ZnONF with crystalline planes of (100), (002), (101), (102), (110), (103), (200), (112), (201), (004), and (202), respectively. This result suggests that ZnONF/GO was successfully composited.

**Figure 4:**
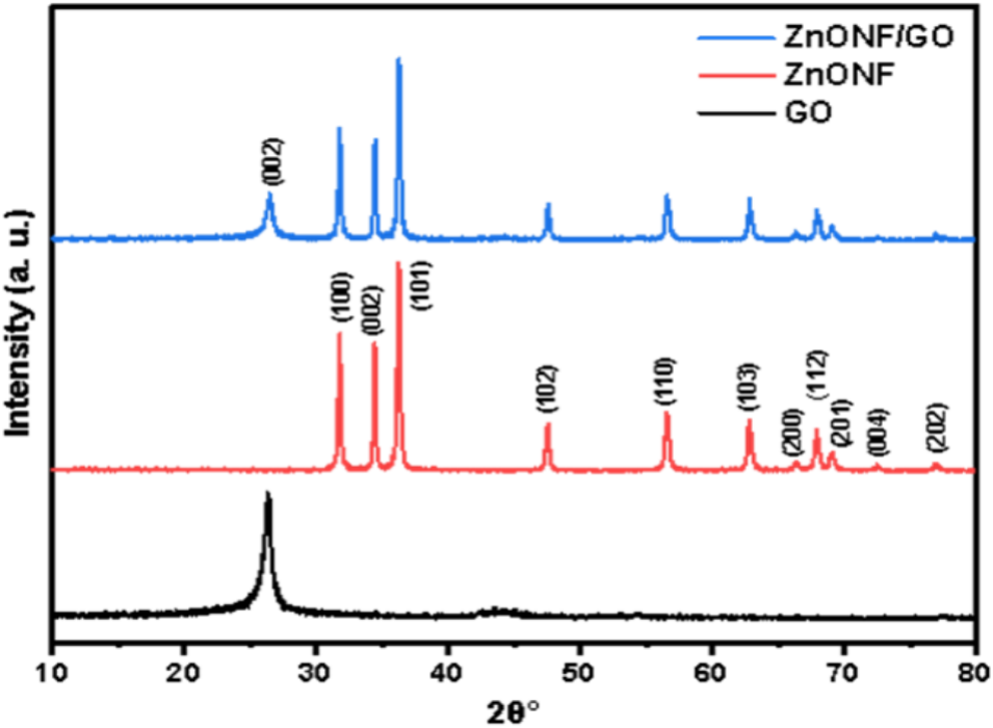
XRD pattern of ZnONF, GO, and ZnONF/GO.

The FESEM measurements were carried out to examine the morphology of ZnONF powder synthesized using the hydrothermal method at 160 °C, as shown in Figure 5a–c at different magnifications. The obtained morphology consists of nanoflowers with several nanoflakes on the petals. The histogram data of the flake size distribution (Figure 5d) have an average of 62.33 nm. The mechanism of ZnONF formation starts with the formation of nuclei, namely, the nucleation process, followed by growth. During the growth process, the crystals form branches or petals, resulting in the growth of ZnONF.^45^

**Figure 5:**
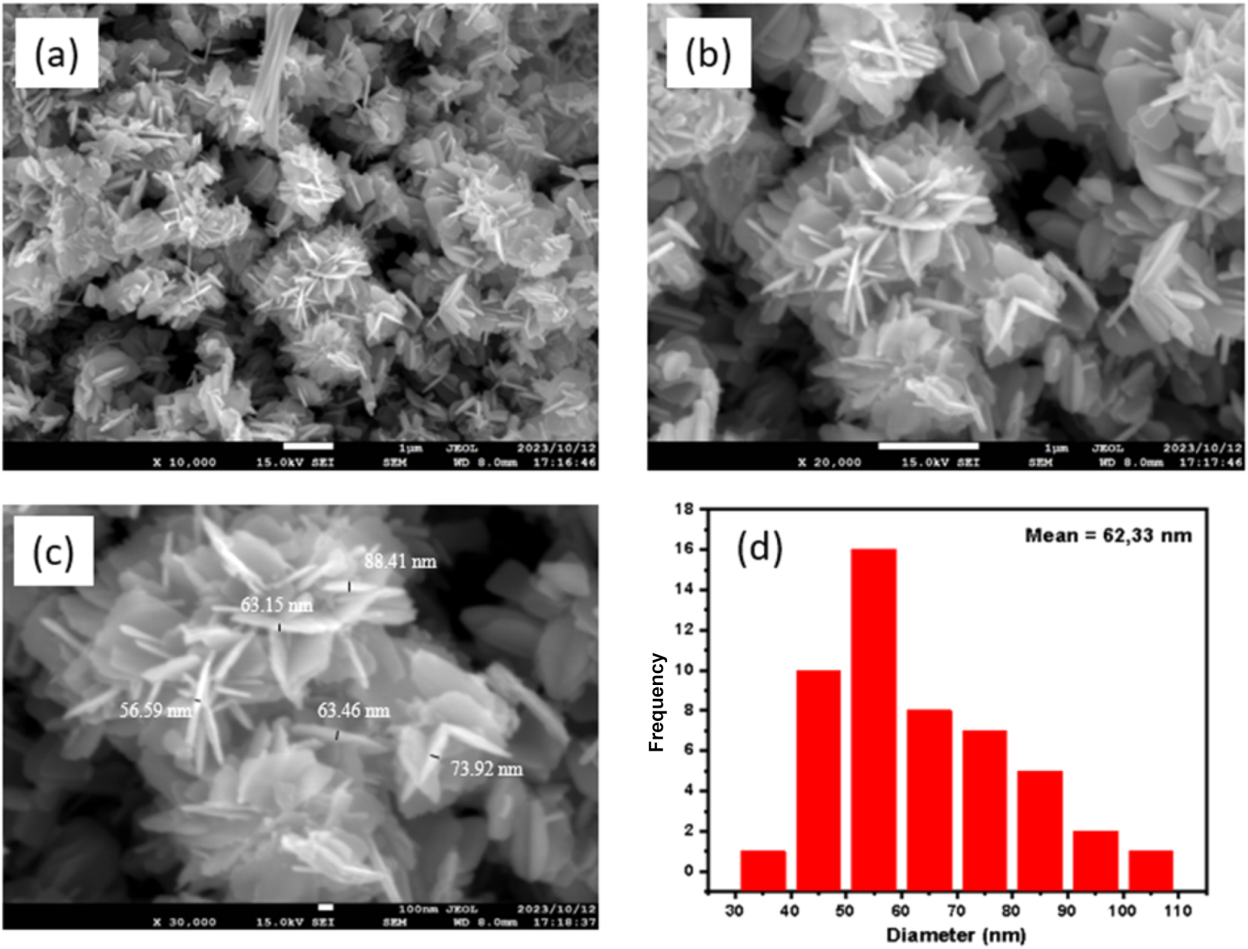
FESEM on ZnONF sample with different magnifications of (a) 10,000×, (b) 20,000×, (c) 30,000×, and (d) histogram data of ZnONF petal size distribution.

The morphological states of ZnONF typically show many petals, providing a larger surface area. The increased surface area offers larger active sites for the adsorption of the analyte molecules. ZnONF is more sensitive for analyte detection compared to the other ZnO nanoparticle morphologies.^25^ Therefore, ZnONF has potential performance in the field of electrochemical sensors, particularly for detecting theophylline.

TEM and HRTEM analyses are performed to determine the crystal structure and morphology of a material. Figure 6a,b shows TEM images of the ZnONF sample in the form of nanoflowers at magnifications of 5,000× and 25,000×, respectively. The 25,000× magnification provides a clearer TEM image showing the arrangement of each nanoflower composed of several petals. Meanwhile, the HRTEM image with a magnification of 5,00,000× (Figure 6c, inset) shows the lattice fringes corresponding to ZnONF lattice, with the inset displaying a *d*-spacing value of 0.26 nm between two lattice fringes, which corresponds to the (002) plane of the hexagonal phase of ZnONF as confirmed by XRD results. The crystalline nature of ZnO was also confirmed by selected area electron diffraction (SAED) as illustrated in Figure 6d, inset, showing an atomic pattern *a* = *b* < *c* consistent with the wurtzite crystal structure of ZnONF.

**Figure 6:**
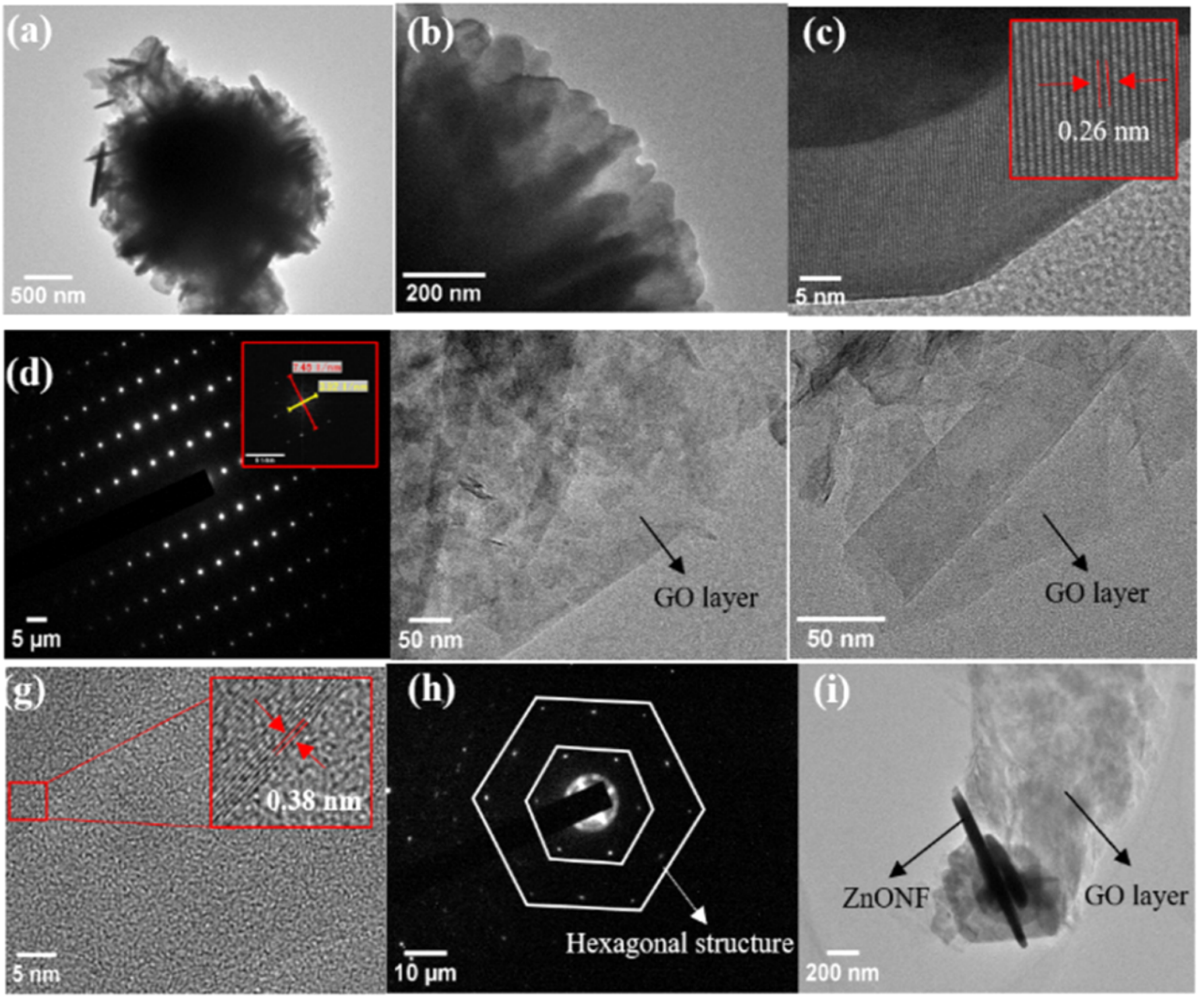
TEM and HRTEM images of (a–d) ZnONF, (e–h) GO, and (i) ZnONF/GO.

The TEM micrograph of GO is shown in Figure 6e,f with a magnification of 50,000× and 1,00,000×. The morphology of GO is shown as several overlapping thin transparent sheets, with the sheets folding over each other.^46^ The HRTEM image with a magnification of 5,00,000× (Figure 6g, inset) shows the lattice fringes corresponding to GO. The inset shows a *d*-spacing value of 0.38 nm between two lattice fringes, which corresponds to the hkl (002) plane, validating the XRD analysis results in Figure 4. The SAED pattern in Figure 6h confirms the crystalline nature of GO with a hexagonal crystal structure. The success of preparing the nanocomposite from the ZnONF/GO sample was evidenced by the TEM micrograph at a magnification of 10,000× in Figure 6i. The ZnONF in the form of nanoflowers is present on the surface of the thin GO sheets. This indicates that the nanocomposite with the GO material has been successfully synthesized.

### Characterization of the Electrode

Topographic results from the modification of SPE using GO, BDDNPs, GO/BDDNPs, and ZnONF/GO/BDDNPs were obtained through the SEM-EDX characterization. All samples were examined at a magnification of 5000×, as shown in Figure 7a,c,e,g,i. The bare SPE, depicted in Figure 7a, shows a smooth, unmodified SPE surface. Figure 7c shows that SPE was successfully modified with the GO sheet, which is further confirmed by the EDX measurement (Figure 7d). Figure 7e shows the successful modification, evidenced by the uniform distribution of BDDNPs across the SPE surface. Furthermore, the EDX results for the BDDNPs sample (Figure 7f) show an increase in the carbon elemental percentage to 97.62%. Additionally, the characterization of BDDNPs using FESEM, XRD, FTIR, and UV–vis has been previously reported.^15^ When the sample was modified with GO/BDDNPs, as depicted in Figure 7g, the previously evenly distributed BDDNPs were coated with GO sheets, as corroborated by the presence of 6.37% oxygen in Figure 7h. The ZnONF/GO/BDDNPs sample (Figure 7i) also successfully modified the SPE surface, indicated by BDDNPs coated with GO sheets and the presence of ZnONF scattered on the GO surface. The successful modification is further supported by the EDX data, showing elements C, O, and Zn, as shown in Figure 7j. The distribution of C, O, and Zn elements through EDX mapping is shown in Figure S2.

**Figure 7:**
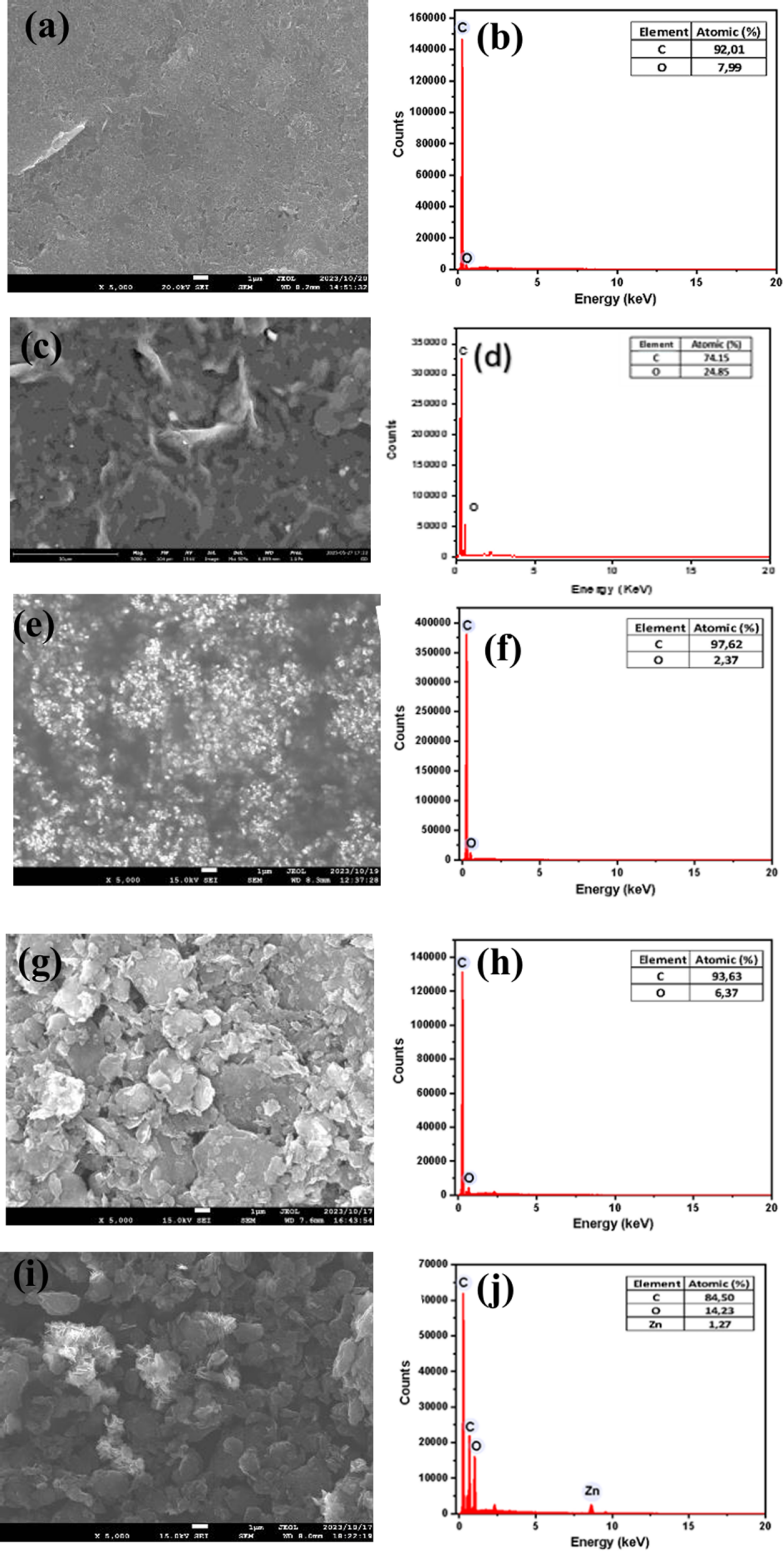
SEM-EDX with 5000× magnification on the electrodes: (a, b) bare SPE, (c, d) GO/SPE, (e, f) BDDNPs/SPE, (g, h) GO/BDDNPs/SPE, (i, j) ZnONF/GO/BDDNPs/SPE.

The electrochemical study of the modified electrodes was analyzed by using EIS and *C–V* methods. EIS analysis was performed to investigate the charge transfer between the electrode surface and the electrolyte. This method provides insights into the electrochemical properties of the modified electrodes and can be applied to evaluate the stepwise sensor fabrication process.^47^ The frequency range of 0.1 Hz to 100 kHz was kept constant for all EIS operations.

The Nyquist impedance plot for BDDNPs, GO/BDDNPs, and ZnONF/GO/BDDNPs in 5 mM K_3_[Fe­(CN)_6_] combined with 0.1 M KCl is shown in Figure 8. The inset shows the Randles circuit model, which includes cell resistance (*R*_Ω_), charge transfer resistance (*R*_ct_), Warburg diffusion process (*Z*_w_), and a constant phase element (CPE). The *R*_ct_ value was estimated based on the difference between Z′ and −Z″. A smaller *R*_ct_ value indicates higher electrocatalytic activity.^2^ The *R*_ct_ values for bare, GO, BDDNPs, GO/BDDNPs, and ZnONF/GO/BDDNPs were 14,000, 1750, 3000, 1400, and 400 Ω, respectively. Based on the results, the ZnONF/GO/BDDNPs electrode has the smallest *R*_ct_ value, signifying a superior charge transfer rate. The flower-like ZnONF morphology plays a critical role in enhancing the charge transfer rate by providing a high catalytic surface area. The *R*_ct_ value correlated with the active surface area of the electrode, which was further verified using *C–V* analysis, as shown in Figure S3.

**Figure 8:**
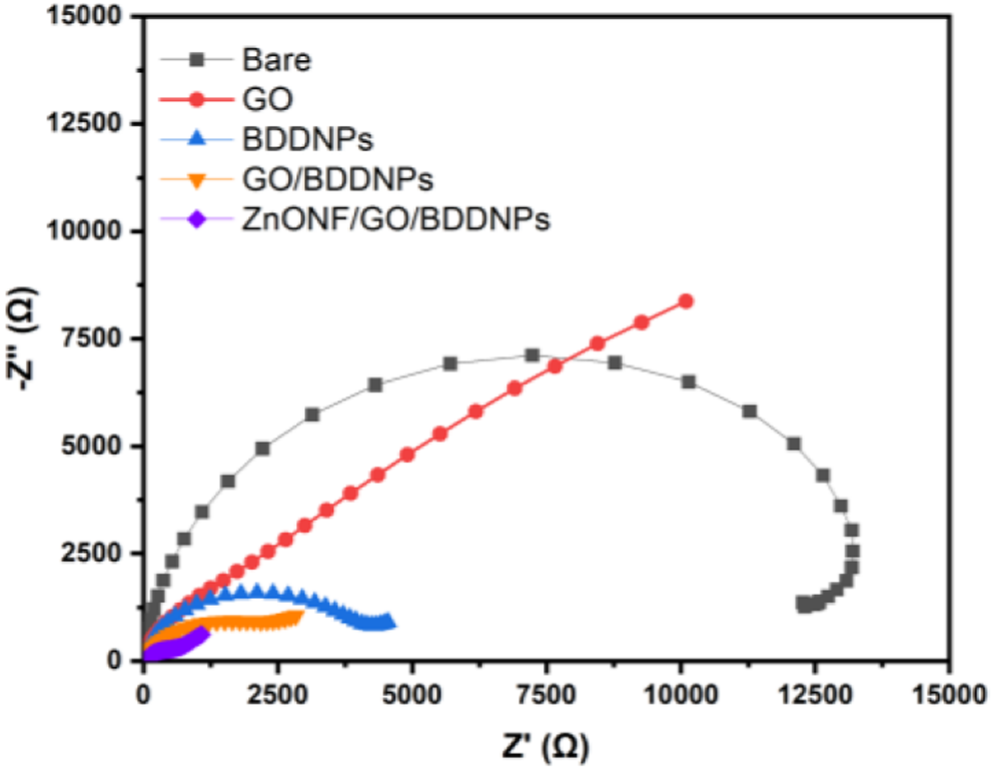
Nyquist graph of bare, GO, BDDNPs, GO/BDDNPs, and ZnONF/GO/BDDNPs.

The electrochemically active surface area of ZnONF/GO/BDDNPs was studied using a 5 mM Fe­(CN_6_)^3‑/4‑^ system in combination with 0.1 M KCl as shown in Figure S3. The Randles-Sevcik equation was used for the calculation as follows:^48^

$$I_{p}=\left(2.69\times 10^{5}\right)\times n^{3/2}\times D^{1/2}\times v^{1/2}\times A\times C$$

where *I_p_* is the peak current (A), n is the number of electrons transferred, D is the diffusion coefficient of *K*_3_[Fe­(CN)_6_] (7.6 × 10–6 cm^2^/s), v is the scanning rate, *A* is the electrochemically active surface area (cm^2^), and *C* is the concentration of bulk solution (5 mM). The linear plot slope square root of the scan rate against the peak current was used to obtain the electrochemically active surface area. Based on the results, the electrochemically active surface area values for bare, GO, BDDNPs, GO/BDDNPs, and ZnONF/GO/BDDNPs were estimated to be 0.0333, 0.0064, 0.0296, 0.0361, and 0.045 cm^2^, respectively. This shows that ZnONF/GO/BDDNPs have a superior electrocatalytic potential compared to the bare, GO, BDDNPs, and GO/BDDNPs. The results are in good agreement with those obtained from the EIS analysis.

The BDDNPs, GO/BDDNPs, and ZnONF/GO/BDDNPs electrodes were also subjected to *C–V* measurements for the determination of theophylline. As shown in Figure S4, only the oxidation peak of theophylline was present, with no reduction peak current observed during the reverse scan. This indicates that the oxidation of theophylline is irreversible, and the oxidation peak of theophylline was observed at a potential of +0.84 V (V vs Ag/AgCl). The peak currents were 7.48 μA, 22.24 μA, and 31.80 μA for the BDDNPs, GO/BDDNPs, and ZnONF/GO/BDDNPs electrodes, respectively. These results show that the ZnONF/GO/BDDNPs electrode had the highest peak current among the modifications, indicating superior electrocatalytic activity.

### Electrochemical Study

The signal to background (S/B) measurement was used to determine the ratio between the background current and the analyte current to be detected.^48^ The greater the ratio, the higher the S/B value, indicating that the electrode used is more sensitive and capable of measuring the analyte at lower concentrations. Figure 9a–c shows the S/B measurement results on the ZnONF, GO, ZnONF/GO, and ZnONF/BDDNPs electrodes to determine the synergistic effect on each electrode modification. S/B values of 2.91, 3.20, 3.26, and 3.39 were obtained for the electrodes, respectively. Figure 9d–f shows that the BDDNPs/SPE, GO/BDDNPs/SPE, and ZnONF/GO/BDDNPs/SPE electrodes detected theophylline at potentials of +0.80, +0.75, and +0.73 V, respectively. A shift to a more negative potential indicates that the ZnONF/GO/BDDNPs electrode is becoming more sensitive. Furthermore, the ZnONF/GO/BDDNPs/SPE electrode produced a higher S/B value of 4.91 compared to those of the BDDNPs/SPE and GO/BDDNPs/SPE, with values of 2.81 and 3.26, respectively. ZnONF/GO/BDDNPs/SPE had the highest S/B value, resulting in high sensitivity; hence, only this electrode was used in subsequent measurements.

**Figure 9:**
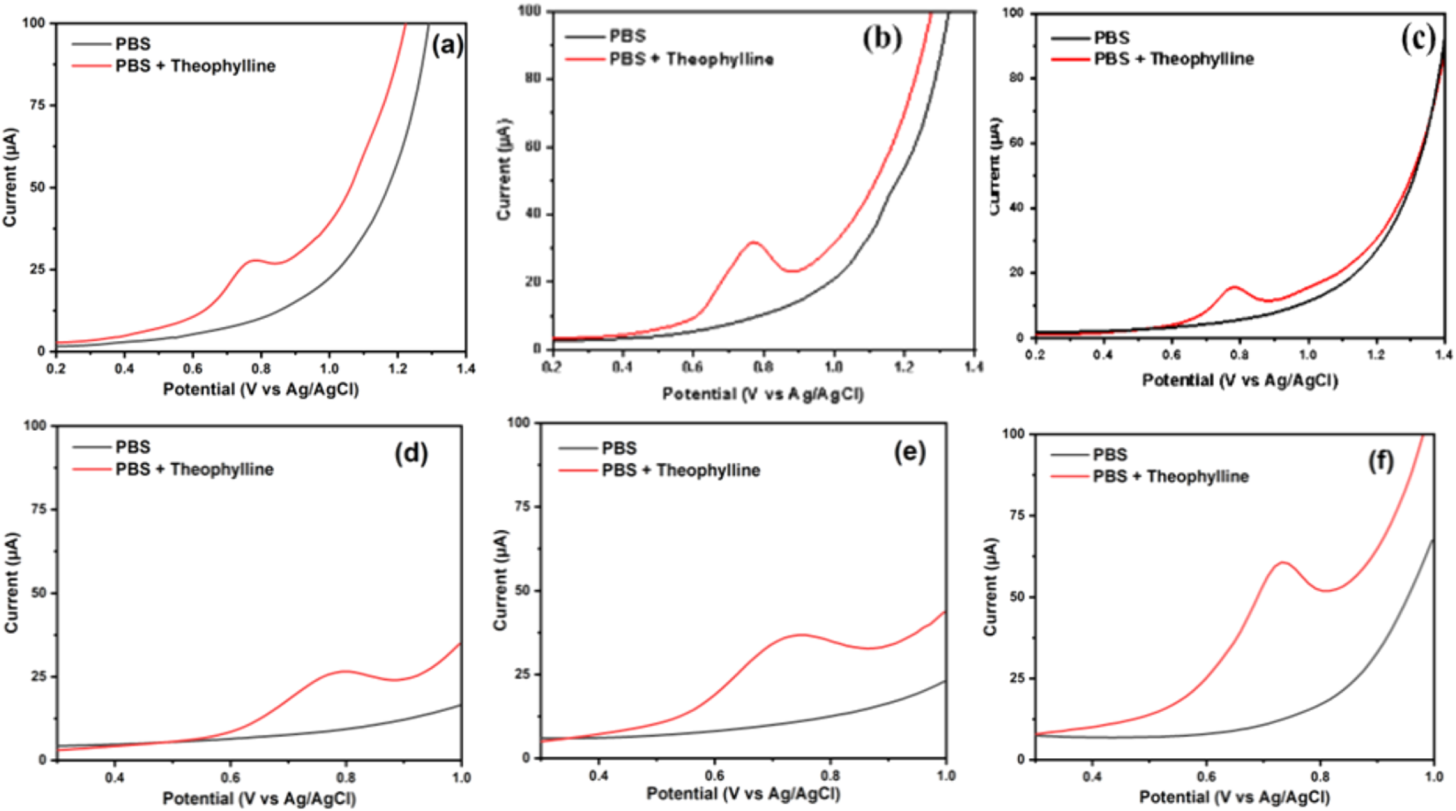
DPV curves for S/B determination in theophylline 120 μM measurement in PBS pH 7 using electrodes: (a) bare, (b) GO, (c) ZnONFs, (d) BDDNPs, (e) GO/BDDNPs, (f) ZnONF/GO/BDDNPs.

In electrochemical measurements, the supporting electrolyte solution with varying pH values plays a crucial role. Therefore, it is essential to investigate the influence of electrolyte pH on theophylline to determine the optimal pH. A pH study was also conducted to examine the effect on the current and potential during the oxidation of theophylline. The variation of pH on 0.1 M PBS used (pH 5 to 9) applied to the ZnONF/GO/BDDNPs electrode is shown in Figure 10a. As the pH increases, a shift to more negative potentials was observed (Figure 10b), indicating the role of protons in the electrochemical oxidation process.^1^ The proposed detection mechanism, shown in Figure 11, entails the transfer of two protons and two electrons during the oxidation of theophylline, which influences the resulting optimal pH value. The highest peak current was observed at a pH value of 7; hence, this pH was optimized for further investigation.

**Figure 10:**
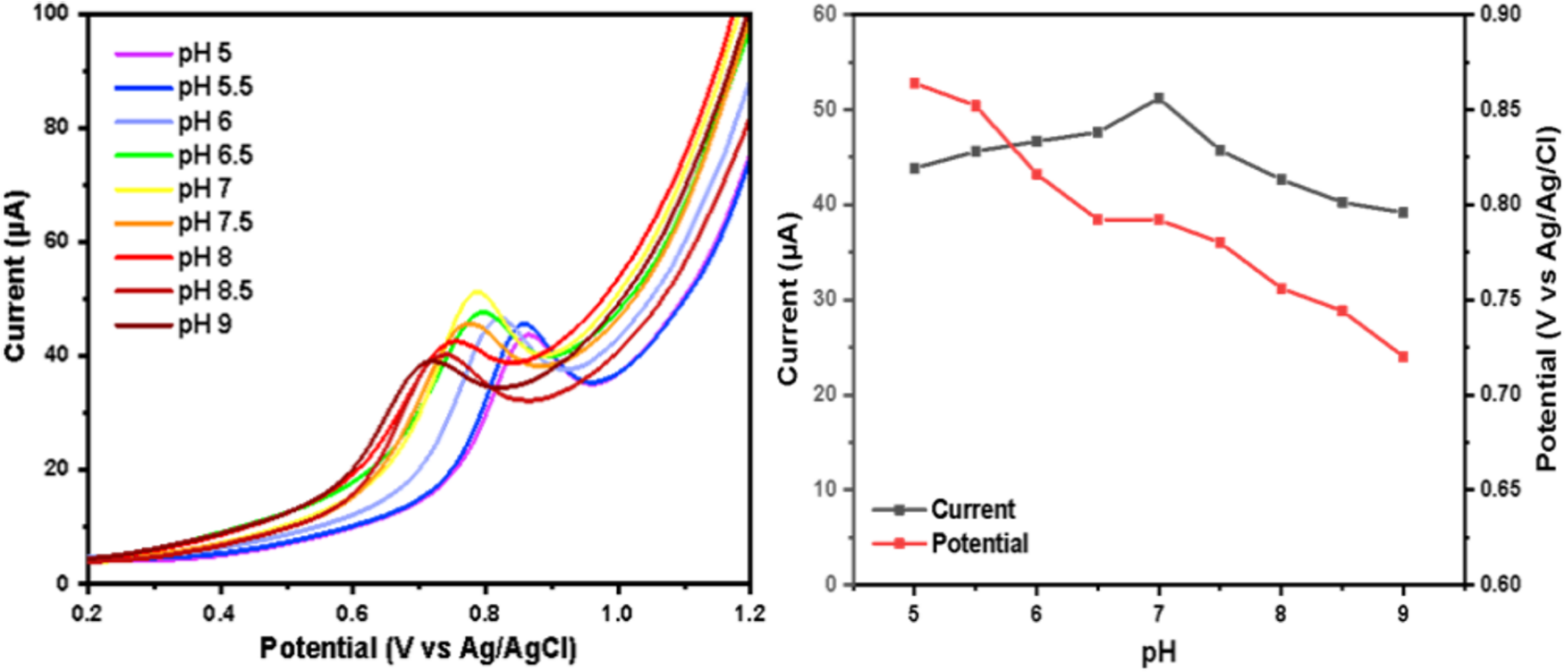
(a) DPV curves at various pHs. (b) Graph of the relationship between pH, potential, and current using ZnONF/GO/BDDNPs.

**Figure 11:**
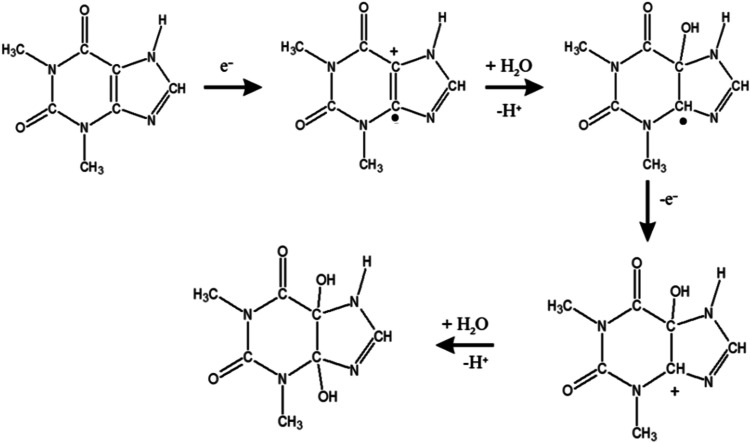
Possible mechanism of theophylline oxidation.

The DPV response was recorded on the ZnONF/GO/BDDNPs electrode using various theophylline concentrations ranging from 50 to 120 μM in PBS at pH 7 for linearity measurements with three repetitions. The concentration variations used were based on the range that can cause side effects in the body, namely, 55–111 μM. The results showed that the measured anodic peak current increased with higher theophylline concentrations, as shown in Figure 12.

**Figure 12:**
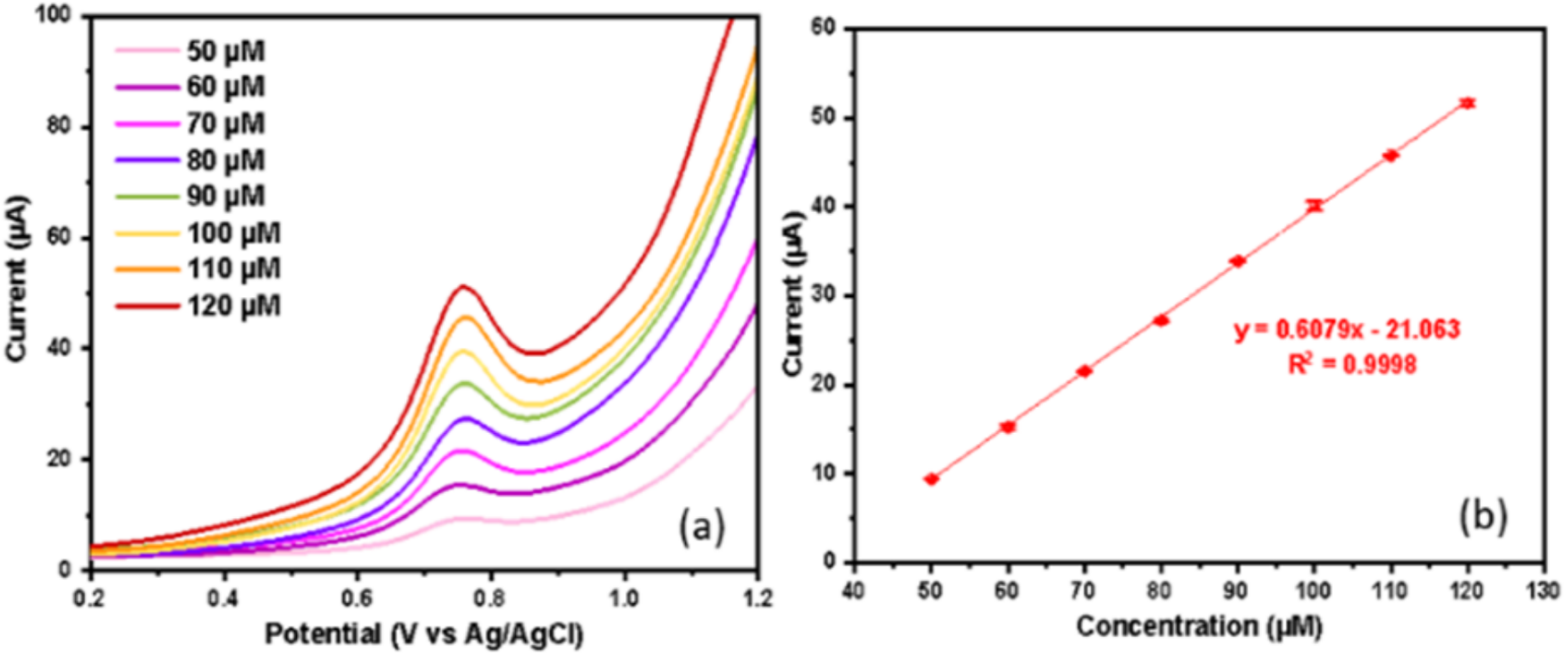
DPV curves. (a) Linearity measurement. (b) Calibration curve between concentration and current for theophylline 50–120 μM measurement in PBS pH 7 using ZnONF/GO/BDDNPs electrode.

A calibration curve between the peak current values and the theophylline concentration with error bars corresponding to the standard deviation was created for the limit of detection (LOD) and limit of quantitation (LOQ) calculations. The ZnONF/GO/BDDNPs/SPE electrochemical sensor for theophylline had good LOD and LOQ values of 0.17 and 0.58 μM, respectively. The equations used were LOD = 3s/m and LOQ = 10s/m, where s and m represent the standard deviation and the slope of the calibration plot, respectively.^1^ The performance of ZnONF/GO/BDDNPs was compared with other recent studies, as shown in Table 1. Compared to previous studies, the LOD value obtained was quite low, allowing for the detection of even the smallest theophylline concentrations. Therefore, it can be concluded that the ZnONF/GO/BDDNPs/SPE electrode is suitable for the detection of theophylline.

**Table 1:** Comparison of Electrochemical Sensors for Theophylline Detection with Previous Studies

electrode	linear range (μM)	LOD (μM)	references
BDD	30–100	4.58	^48^
BDD/NiNP		2.79	
MWNTs/Au/poly-l-lysine/SPE	10–200	2.00	^49^
TiO_2_NRs/MWCNT/GCE	1- 893	0.56	^50^
Poly(H-A)/GCE	0.4–17	0.32	^51^
PSA/CNF/GCE	0.6–137	0.2	^52^
DP-Py COF/AuNPs/GCE	0.9–20	0.19	^53^
GQD/SPE	1–700	0.2	^54^
CNOs/GCE	5.16–108.25	0.35	^55^
ZnONF/GO/BDDNPs/SPE	50–120	0.17	this work

Selectivity measurements for theophylline on the ZnONF/GO/BDDNPs electrode were conducted using interfering compounds, including theobromine, caffeine, ascorbic acid, and uric acid, as shown in Figure 13. These solutions have the potential to interfere with the analytical performance as both are methylxanthine compounds with structures similar to theophylline and determination in the real sample. Measurements were carried out by adding theophylline and the interfering compounds in a 1:1 v/v ratio with each compound at a concentration of 120 μM. When the analyte and interfering compounds were measured simultaneously, there was a lower peak and an insignificant shift. However, the ZnONF/GO/BDDNPs electrode can still be considered selective because it produced two distinct peaks. This implies that the electrode is selective for analyzing theophylline when measured with similar compounds and determined in the real sample.

**Figure 13:**
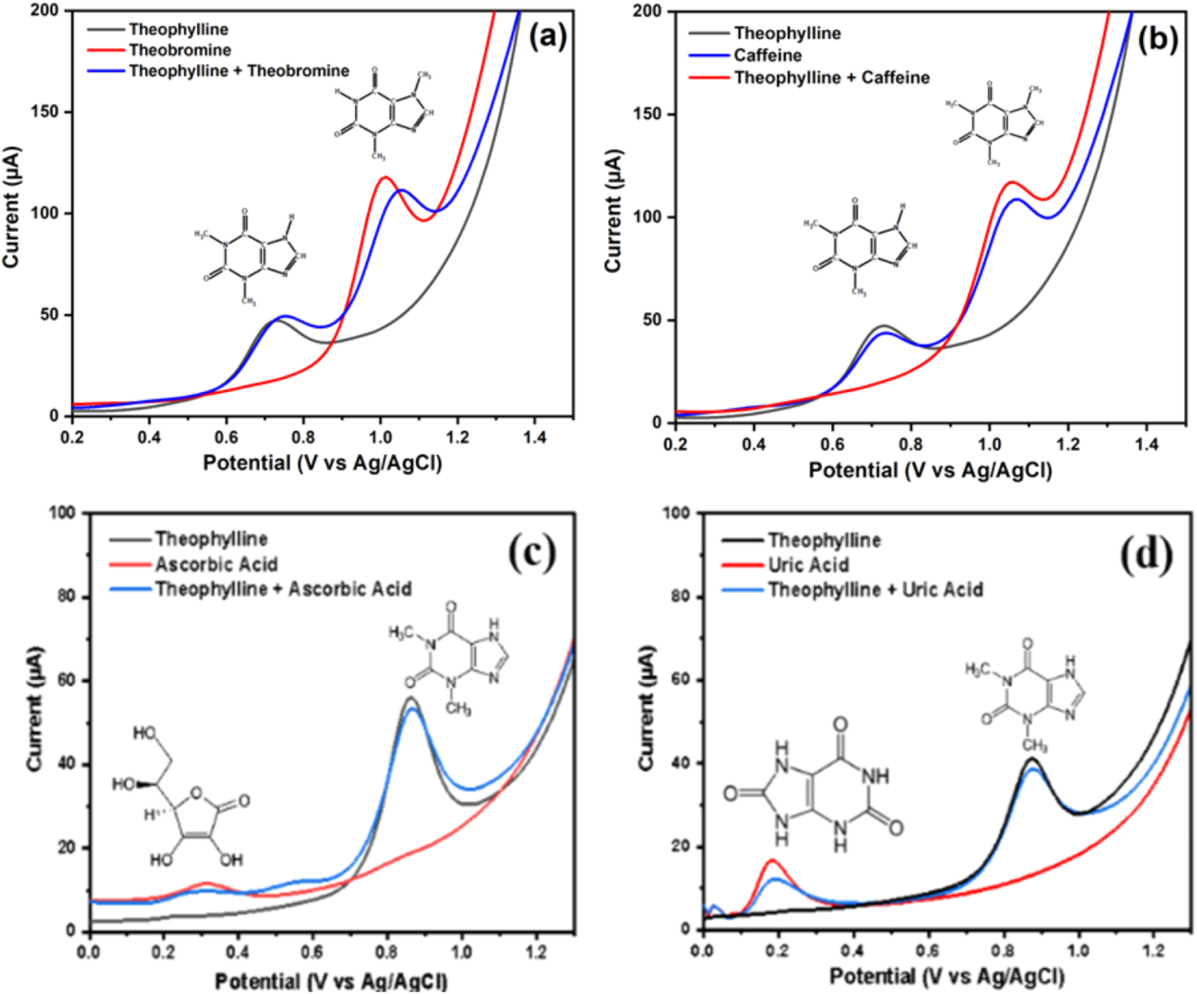
DPV curves for selectivity measurement: (a) Theobromine, (b) caffeine, (c) ascorbic acid, and (d) uric acid using the ZnONF/GO/BDDNPs electrode.

Reproducibility determination was used to measure the precision and stability of the ZnONF/GO/BDDNPs electrode with theophylline as the analyte, measured 30 times over different days. To confirm the optimal stability, further observations were conducted over 30 days, as shown in Figure S5. Throughout the 30 days of measurement, there were no significant changes in the generated peak current. The data obtained were calculated and yielded a %RSD of 1.06%. This value, less than 2%, indicates that the ZnONF/GO/BDDNPs electrode has excellent precision and stability.

Recovery measurement is used to assess the accuracy of an electrode in an analysis. In this study, the ZnONF/GO/BDDNPs electrode was tested on real samples, including urine, coffee, and a commercial drug in the form of a theophylline capsule branded Theobron. All measurements were spiked with the addition of 120 μM theophylline except for the theophylline capsule sample. After measurement, the three real samples showed anodic peaks with % Recovery values for urine, coffee, and the drug being 103.69, 96.2, and 94.19%, respectively. More detailed data from the measurements are presented in Table 2. These results imply that the ZnONFs/GO/BDDNPs electrode can be recommended for use in real samples due to its good accuracy.

**Table 2:** Recovery Percentage of the ZnONF/GO/BDDNPs Electrode in Measuring 120 μM Theophylline Tested on Real Samples

sample	added concentration (μM)	found concentration (μM)	% recovery
urine	120	127.18	103.69% ± 1.45
drug	0	118.62	96.2% ± 0.83
coffee	120	116.33	94.19% ± 2.72

## Conclusions

In conclusion, this study successfully used a novel electrochemical biosensor based on ZnONF/GO/BDDNPs modified on SPE for the detection of theophylline. SEM-EDX characterization confirmed that ZnONF/GO/BDDNPs modification was successfully implemented on the SPE surface. ZnONF/GO/BDDNPs have good electrocatalytic activity, as evidenced by EIS and *C–V* analysis. The developed electrode had high sensitivity, as indicated by an S/B value of 4.91. It also achieved a good limit of detection (0.17 μM) and a limit of quantification (0.58 μM), with a linear range of 50–120 μM compared with other reported biosensors. The reproducibility test demonstrated good precision with a %RSD value of 1.06%. Additionally, the electrode showed good selectivity for similar compounds in the real sample, such as theobromine, caffeine, uric acid, and ascorbic acid. The ZnONF/GO/BDDNPs electrode was successfully used for the accurate detection of theophylline in real samples, specifically urine, coffee, and commercial drugs, with % Recovery values of 103.69%, 96.2%, and 94.19%, respectively. The developed biosensor demonstrates good electrocatalytic activity, sensitivity, selectivity, reproducibility, stability, accuracy, and detection limits compared to those of others previously reported. The proposed method is easy, simple, and accurate, making the ZnONF/GO/BDDNPs electrode suitable for qualitative and quantitative analysis in future theophylline measurements. Further studies may combine other materials, such as integrated microneedles, for in vitro applications.

## Supplementary Material



## Notes

Additional material characterization, UV–vis and EDX mapping, *C–V* curves for various scan rates, *C–V* curves of several electrodes, S/B, and reproducibility test (PDF)
