# Supplementary material for: Sensitive and Selective Theophylline Electrochemical Detection on ZnO Nanoflowers/Graphene Oxide/Boron-Doped Diamond Nanoparticles
Source: ACS Omega. 2025 Jun 20;10(25):27435–47. doi: 10.1021/acsomega.5c03180 (PMC12224099; doi:10.1021/acsomega.5c03180)
Supplement: Supplementary file 1 [file ao5c03180_si_001.pdf]

Supplementary Data

Sensitive and Selective Theophylline Electrochemical Detection on ZnO Nanoflowers/Graphene Oxide/Boron-Doped Diamond Nanoparticles

Syarifa Sabilla[1], Prastika Krisma Jiwanti[1,2*], Takeshi Kondo[3], Rena Akiyama[3], Taisuke Kusunoki[3], Afif Akmal Afkauni[4], Anisa Sufia Latifah[4], Arramel[4], Tahta Amrillah[1,2], Yeni Wahyuni Hartati[5], Qonita Kurnia Anjani[6], Yulia Mariana Tesa Ayudia Putri[7], Jarnuzi Gunlazuardi[7]

[1]Nanotechnology Engineering, Faculty of Advanced Technology and Multidiscipline, Universitas Airlangga, Surabaya 60115, Indonesia

[2]Airlangga Functional Nanomaterials Research Group, Faculty of Advanced Technology and Multidiscipline, Universitas Airlangga, Surabaya 60115, Indonesia

[3]Department of Pure and Applied Chemistry, Tokyo University of Science, 2641 Yamazaki, Noda 278-8510, Chiba, Japan

[4]Center of Excellence Applied Physics and Chemistry, Nano Center Indonesia, South Tangerang, Banten 15314, Indonesia

[5]Department of Chemistry, Faculty of Mathematics and Natural Sciences, Universitas Padjadjaran, Jatinangor 45363, Indonesia

[6]School of Pharmacy, Medical Biology Centre, Queen's University Belfast, 97 Lisburn Road, Belfast BT9 7BL, UK

[7]Department of Chemistry, Faculty of Mathematics and Natural Sciences, Universitas Indonesia, Kampus UI Depok, Jakarta 16424, Indonesia

(*) Corresponding author. prastika.krisma@ftmm.unair.ac.id

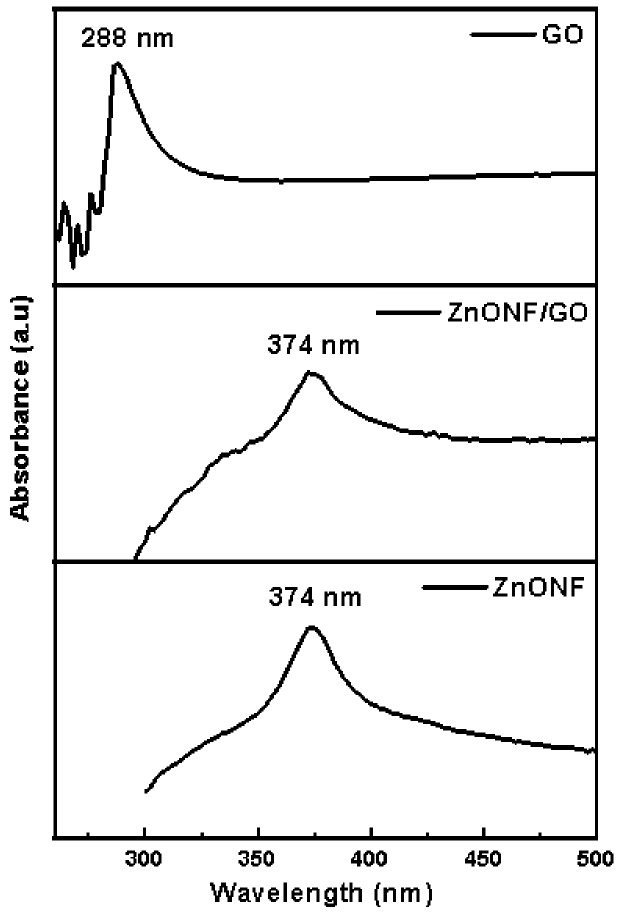

Figure S1. UV-Vis spectra of ZnONF, GO, and ZnONF/GO

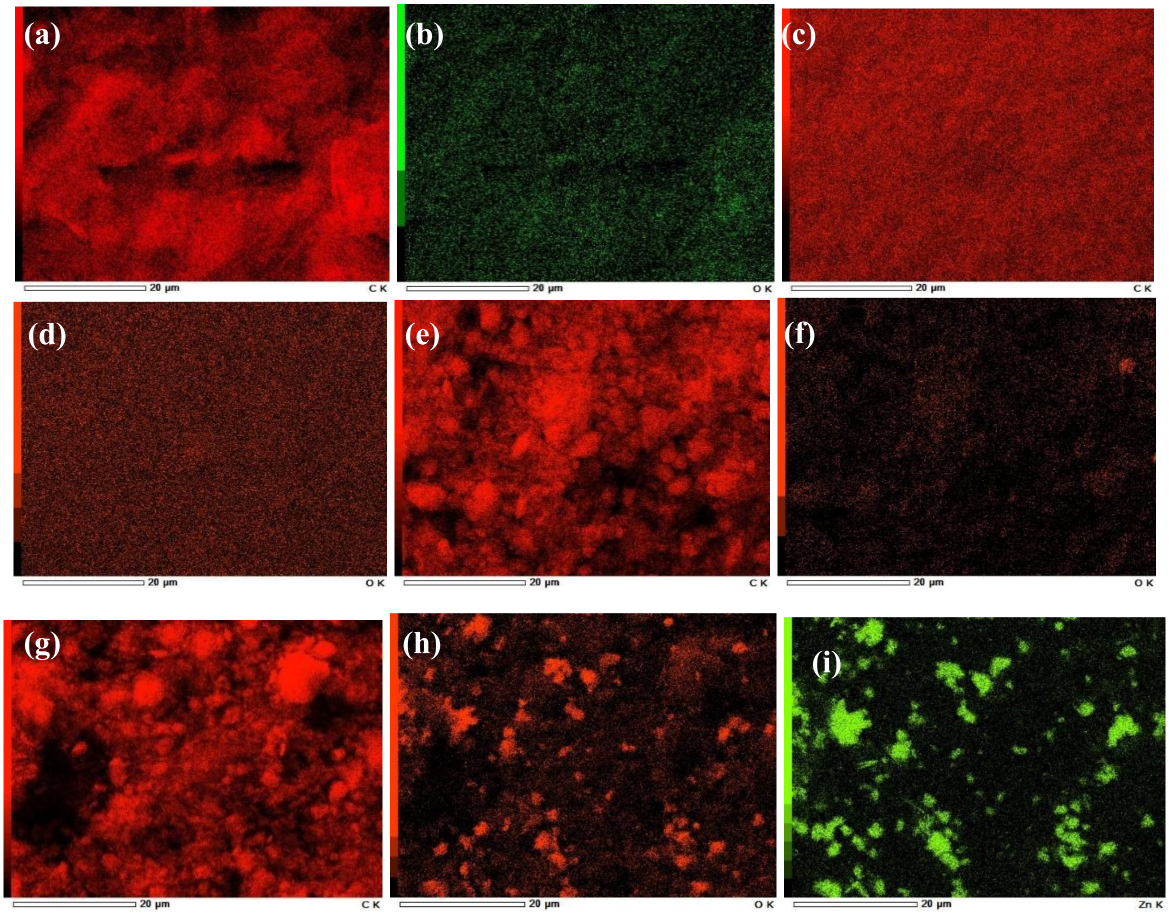

Figure S2. EDX mapping (a-b) bare SPE (c-d) BDDNPs/SPE (e-f) GO/BDDNPs/SPE (g-i) ZnO/GO/BDDNPs/SPE

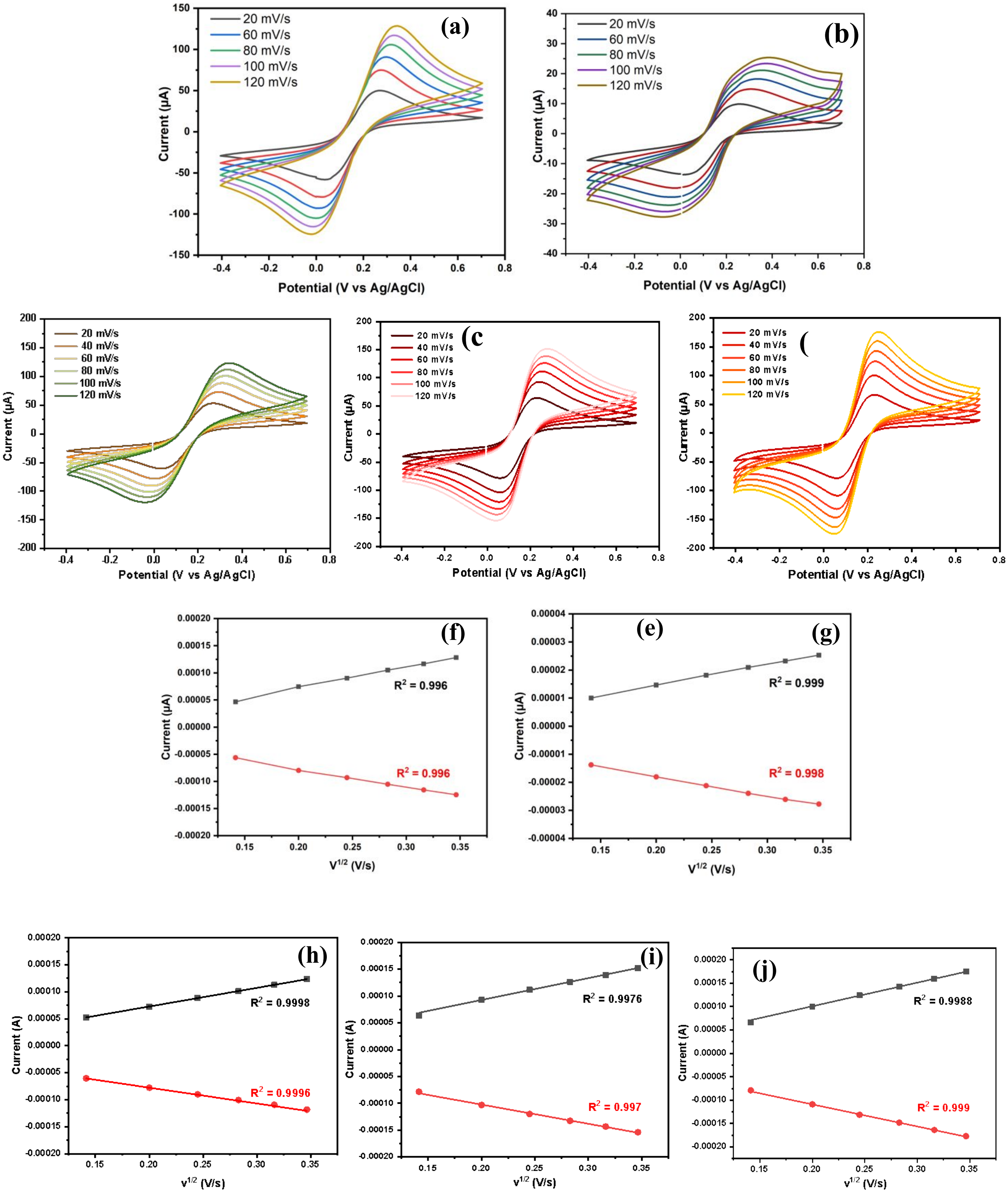

Figure S3. CV curves for various scan rates from 20 to120 mV/s measured in 5 Mm $K_3[Fe(CN)_6]$ dissolved in 0.1 M KCl solution and A plot of the square root of the scan rate vs peak oxidation and reduction current using Bare (a and f) GO (b and g) BDDNPs (c and h) GO/BDDNPs (d and i) ZnONF/ GO/BDDNPs (e and j)

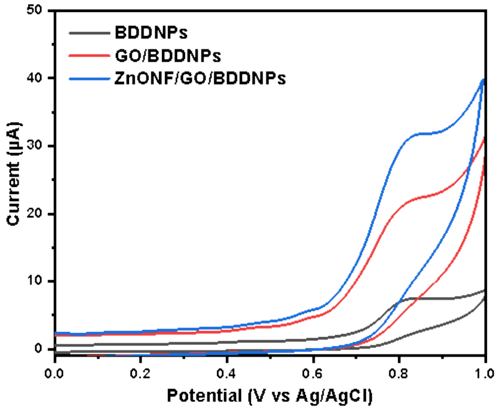

Figure S4. CV curves for BDDNPs, GO/BDDNPs, dan ZnONF/GO/BDDNPs electrode in 120 μM theophylline at a scan rate 50 mV/s

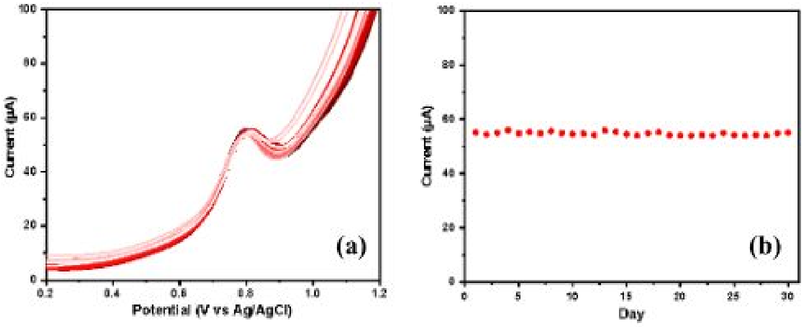

Figure S5. DPV curves (a) Reproducibility measurement (b) Graph of Days vs. Current for Theophylline 120 µM Measurement in PBS pH 7 using ZnONF/GO/BDDNPs Electrode
